# Dmp1α Inhibits HER2/neu-Induced Mammary Tumorigenesis

**DOI:** 10.1371/journal.pone.0077870

**Published:** 2013-10-29

**Authors:** Elizabeth A. Fry, Pankaj Taneja, Dejan Maglic, Sinan Zhu, Guangchao Sui, Kazushi Inoue

**Affiliations:** 1 Department of Pathology, Wake Forest University Health Sciences, Winston-Salem, North Carolina, United States of America; 2 Department of Cancer Biology, Wake Forest University Health Sciences, Winston-Salem, North Carolina, United States of America; 3 Graduate Program in Molecular Medicine, Wake Forest University Health Sciences, Winston-Salem, North Carolina, United States of America; University of Alabama at Birmingham, United States of America

## Abstract

Our recent study shows a pivotal role of Dmp1 in quenching hyperproliferative signals from HER2 to the Arf-p53 pathway as a safety mechanism to prevent breast carcinogenesis. To directly demonstrate the role of Dmp1 in preventing HER2/neu-driven oncogenic transformation, we established *Flag-Dmp1α* transgenic mice (*MDTG*) under the control of the mouse mammary tumor virus (*MMTV*) promoter. The mice were viable but exhibited poorly developed mammary glands with markedly reduced milk production; thus more than half of parous females were unable to support the lives of new born pups. The mammary glands of the MDTG mice had very low Ki-67 expression but high levels of Arf, Ink4a, p53, and p21^Cip1^, markers of senescence and accelerated aging. In all strains of generated *MDTG;neu* mice, tumor development was significantly delayed with decreased tumor weight. Tumors from *MDTG;neu* mice expressed Flag-Dmp1α and Ki-67 in a mutually exclusive fashion indicating that transgenic *Dmp1α* prevented tumor growth *in vivo*. Genomic DNA analyses showed that the *Dmp1α* transgene was partially lost in half of the *MDTG;neu* tumors, and Western blot analyses showed Dmp1α protein downregulation in 80% of the cases. Our data demonstrate critical roles of Dmp1 in preventing mammary tumorigenesis and raise the possibility of treating breast cancer by restoring Dmp1α expression.

## Introduction

Breast cancer is one of the most important public health issues in the United States and most industrialized countries [1]–[4]. In the U.S., an estimated 210,000 women will be diagnosed with breast cancer in 2013 [2], making it the most common cancer in U.S. women, and second only to lung cancer in cancer-related death. Approximately 70% of human breast tumors express hormone receptors, the estrogen receptor (ER) and/or progesterone receptor (PR). ER is the primary transcription factor driving oncogenesis in hormone receptor-positive breast cancers, and thus usually responsive to adjuvant hormonal therapy with anti-estrogens or aromatase inhibitors, giving a more favorable prognosis [1]. Conversely, ER-negative tumors are frequently associated with more aggressive disease with poorer clinical outcomes, including amplification of *HER2* or c-*Myc* oncogenes or gain-of-function mutation of *p53* [1], [4]. *BRCA1/2* are high-penetrance breast cancer predisposition genes identified by genome-wide linkage analysis and positional cloning. Mutations of so-called low penetrance breast cancer genes functionally related to *BRCA1/2,* such as *CHEK2*, *ATM*, *BRIP1*, and *PALB2,* are rare, but confer an intermediate risk of the disease [5].

The c-ErbB2 gene (HER2) has been identified in the human genome 17q21 and encodes a polypeptide with a kinase domain highly homologous to that of the epidermal growth factor (EGF) receptor [6]. The human *c-ErbB2* gene (*HER2*) is an equivalent of the rat *neu* gene, detected in a series of rat neuro/glioblastomas [6]. HER2/neu encodes a receptor-type tyrosine kinase that belongs to the EGFR family [7]–[9]. It is overexpressed in ∼50% of human breast cancers, primarily due to gene amplification (30%) [9], although protein overexpression without gene amplification is also found in ∼20% of breast cancer cases. HER2/neu overexpression is prominent in metastatic lesions, and thus associated with poor clinical outcomes [7]–[9]. It has been shown that phosphatidylinositol-3′-kinase (PI3K) and serine/threonine kinase Akt/protein kinase B play critical roles in oncogenic HER2/neu signaling [10]. Aberrant HER2/neu expression initially causes cell proliferation, but eventually leads to cell cycle arrest or senescence in normal cells to prevent their malignant transformation, in which Dmp1 plays a critical role [11].

A valine to glutamic acid substitution in the trans-membrane domain of a rat *neu* mutant results in the constitutive aggregation and activation of the receptor in the absence of ligand [12] (reviewed in [13]). In human breast cancers overexpressing HER2, the same trans-membrane point mutation of HER2/neu has not been reported, but its activated splicing variants have been reported in tumors [14], [15]. Transgenic mice expressing activated *neu* under the control of *mouse mammary tumor virus* promoter (*MMTV*-*neu*) develop multifocal mammary tumors at a median age of 7 months with high potential of lung metastasis [16]. Mice bearing the wild-type *ErbB2* allele under the control of the *MMTV* promoter (*MMTV-ErbB2*) have also been established [17]. The usefulness of the *MMTV*-driven transgenic mice as models of breast cancer has been emphasized since the detection of *MMTV env*-like sequence in ∼40% of human breast cancers [13], [18]. In contrast to the rapid tumor progression observed in several transgenic strains carrying the activated *neu* transgene, wild-type *neu* expression in the mammary epithelium results in the development of focal mammary tumors with longer latency than those with constitutively active *neu* (8–12 months vs. 6–7 months) [16], [17]. Interestingly, many of the tumor-bearing transgenic mice developed secondary metastatic lesions in lung indicating that wild-type *neu* overexpression can induce metastatic disease after long latency [17].

Dmp1 (a cyclin D binding myb-like protein 1; also called Dmtf1) is a transcription factor originally isolated in a yeast two-hybrid screen of a murine T-lymphocyte library with cyclin D2 as bait [19]. Dmp1 shows its activity as a tumor suppressor by directly binding to the *Arf* promoter to activate its gene expression, and thereby induces p53-dependent cell cycle arrest [20], [21] (reviewed in [22], [23]). Our recent study indicates that Dmp1 directly binds to p53 and neutralizes the negative regulation of p53 by Mdm2, especially in epithelial and hematopoietic cells [24]. *Dmp1*-null murine embryonic fibroblasts (MEFs) give rise to immortalized cell lines that retain wild-type p19^Arf^ and p53, and are transformed by oncogenic Ras alone, suggesting that the activity of the Arf-p53 pathway is significantly subverted in *Dmp1*-deficient cells [25], [26]. The murine *Dmp1* promoter is efficiently activated by oncogenic Ras and HER2 and repressed by E2Fs-mediated mitogenic signals and NF-κB-mediated genotoxic signals [27]–[29], suggesting that the promoter receives both positive and negative regulation.

Both *Dmp1^−/−^* and *Dmp1^+/−^* mice were prone to tumor development when newborn pups were treated with dimethylbenzanthracene or ionizing radiation [25], [26]. Dmp1, p53, and p21^Cip1^ were induced in pre-malignant lesions of *MMTV*-*neu* mice to prevent incipient cancer cells from transformation. Selective *Dmp1* deletion and/or Tbx2/Pokemon overexpression was found in >50% of wild-type HER2/neu carcinomas while the involvement of Arf, Mdm2, or p53 was rare [11]. Tumors induced by the *Eµ*-*Myc*, *K*-*Ras* and *HER2* transgenes were greatly accelerated in both *Dmp1^+/−^* and *Dmp1^−/−^* backgrounds with no differences between groups lacking one or two *Dmp1* alleles, suggesting haploid-insufficiency of *Dmp1* in tumor suppression in these mouse models of human cancers [11], [26], [30]. Mammary tumors from *MMTV-neu*; *Dmp1^+/−^*, *Dmp1^−/−^* mice showed significant downregulation of *Arf* and *p21^Cip1^*, with p53 inactivity and more aggressive phenotypes than tumors with intact *Dmp1* [11].

The h*DMP1* gene is located on chromosome 7q21, a region often deleted in breast cancer and hematopoietic malignancies [31]–[34]. We recently found that loss of heterozygosity (LOH) of h*DMP1* was present in ∼35% of non-small cell lung carcinomas [30], [35], [36]. The h*DMP1* locus encodes at least three splicing variants, i.e. h*DMP1α, β,* and *γ* [32]. The full-length h*DMP1α* gene corresponds to the murine *Dmp1* that positively regulates the Arf-p53 pathway. On the other hand, the hDMP1β and γ isoforms lack the DNA-binding domain, and hDMP1β is dominant-negative for hDMP1α on myeloid differentiation and CD13 induction [32].

To elucidate the role of human *DMP1* (h*DMP1*) in breast cancer, we recently analyzed 110 tumor and normal pairs of human breast cancer samples for the alterations (gene loss or amplification) of the hDMP1-ARF-Hdm2-p53 pathway with follow up of clinical outcomes. LOH of the h*DMP1* locus was found in 42% of human breast cancers, while that of *INK4a/ARF* and *p53* were found in 20% and 34%, respectively. Amplification of the *Hdm2* locus was found in 13% of the samples, which was independent of LOH for h*DMP1*, *INK4a/ARF,* or *p53* [37]. LOH for h*DMP1* was mutually exclusive with that of *INK4a/ARF* and *p53*, and associated with low Ki67 index and diploidy of the nuclear DNA. Consistently, LOH for h*DMP1* was associated with luminal A category and longer relapse-free survival, while that of *p53* was associated with non-luminal A subgroup. Thus, loss of h*DMP1* defined a new disease category with a potential prognostic value for breast cancer patients [37].

In this study, we established *Flag-Dmp1α* transgenic mice (*MDTG*) driven by the *MMTV* promoter to study the effects of Dmp1 expression in mammary gland development, function, and gene/protein expression *in vivo*. We crossed *MDTG* mice with *MMTV-neu* mice to demonstrate the role of Dmp1 in preventing HER2/neu-induced mammary carcinogenesis.

## Materials and Methods

### Creation of *MMTV-Flag-Dmp1α* Mice

To create transgenic mice that constitutively express Dmp1α in mammary glands, we cloned the Flag-tagged murine *Dmp1α* cDNA into the Hind III site of the vector *MMTV-SV40-BSSK* (a gift from Dr. Philip Leder, Harvard Medical School) and created *MMTV-Flag-Dmp1α* transgenic (*MDTG*) mice in the FVB/NJ background using the Transgenic Core of Wake Forest University Health Sciences. The term Flag-Dmp1 indicates Flag-Dmp1α hereafter. Briefly, the transgene construct was microinjected into the pronuclei of fertilized one-cell zygotes from FVB/NJ mice. These zygotes were reimplanted into pseudo-pregnant foster mothers, and the offspring were screened for the presence of the transgene by PCR. Carriers were bred to establish three independent transgenic lines, strains 76, 79, and 138.

### Creation of *MMTV-Flag-Dmp1α;neu (MDTG;neu) bitransgenic* Mice and Whole Mount Preparation of Mammary Glands

One male *MMTV*-*Flag-Dmp1α* -transgenic mouse in each was crossed with two female *MMTV*-*neu* mice to obtain *MDTG;neu* double transgenic mice. We obtained more than 25 *MDTG;neu* bi-transgenic females for each *MDTG* strain. Mice were monitored daily, sacrificed in a CO_2_ chamber when moribund, and tumor tissues were resected from the mice for pathological and molecular genetic analyses. Whole mount sections of wild-type and *MDTG* mammary glands were prepared from 12-week-old nulliparous females as described previously [38].

### Analysis of Mammary Tumors Obtained from the Bi-transgenic Mice

Mice were monitored daily for mammary tumor development. We detected tumors when they reached 3mm in size, and sacrificed the mice 4 weeks after the first tumor was found. After CO_2_ asphyxiation, mammary tumors were dissected from mice to weigh, and then we conducted histopathological and biochemical analyses. All experimental procedures were conducted according to a protocol approved by the Institutional Animal Care and Use Committee of the Wake Forest University Health Sciences.

### Gene Expression Analyses and Gene Copy Number Assay for *Flag-Dmp1α* in Mouse Tails and Tumors

Quantitation of *Flag-Dmp1, p19^Arf^, p16^Ink4a^,* and *p21^Cip1^* mRNAs was conducted by real-time PCR Taqman assay by ABI7500 (Applied Biosystems) using *β-actin* as an internal control (28, 29). The assays for mouse *Arf* (*p19Arf-cDNA*), *Ink4a* (*Ink4a-cDNA-G*), and *β-actin* (*mactbEx3_4*) were custom-designed at ABI. For mouse p21^Cip1^, Mm01303209_m1 was used. Gene copy number assay for *Dmp1* was also performed by real-time PCR aiming at the exons deleted in *Dmp1* knockout mice (*Dmp1 Ex10-11-Ex11*), using *β-actin* (*mbactinex4-ANY*) as an internal control. Tail DNAs from *Dmp1^+/+^*, *Dmp1^+^* ^/−^, and *Dmp1^−/−^* were used as two copies, one copy and zero-copy control.

### Western Blotting

For Western blot analyses of *MDTG;neu* tumors, proteins were extracted with ice-cold EBC buffer with proteinase inhibitors (Calbiochem proteinase inhibitor cocktail III, leupeptin, AEBSF, and aprotinin) [19]. After gel electrophoresis and transfer to nitrocellulose membranes, proteins were visualized by immunoblotting using affinity-purified polyclonal antibodies to Flag-Dmp1 (Abcam ab21536), p53 (sc-6243G), Mdm2 (ab16896, #2A10, Abcam), p19^Arf^ (sc-32748), p16^Ink4a^ (sc-74401), p21^CIP1/WAF1^ (sc-397G), or β-actin (sc-1615, sc-47778), followed by incubation of the filters with horseradish peroxidase–conjugated second antibodies, and reaction with the enhanced chemiluminescence detection kit (Perkin-Elmer).

### Immunohistochemical Staining

Immunohistochemical staining of tissues and tumors were conducted as described previously [28]. The following antibodies were used for immunohistochemistry with formalin-fixed, paraffin-embedded sections: Ki67 (SP6, NeoMarkers), αDDDDK Tag (Abcam ab21536), cleaved caspase 3 (#9661, Cell, Signaling), and HER2 (sc-284, Santa Cruz Biotech).

### Statistical Analyses

Statistical differences of survival in *MMTV-neu* and *MDTG;neu* females (nulliparous) were analyzed by Medcalc software, Mariakerke, Belgium. Statistical analyses for all experiments were conducted using unpaired Student’s *t*-tests.

## Results

### Establishment of *MMTV-Flag-Dmp1α* Mice

To elucidate the roles of Dmp1α in mammary tumor development and prevention of *HER2;neu*-induced mammary carcinogenesis, we created transgenic mice that constitutively express the Flag-tagged murine *Dmp1α* gene under the control of the *MMTV* promoter (*MMTV-Flag-Dmp1α, MDTG*) (Fig. S1; strain 138 results, not shown). Real-time PCR study of the genomic DNA extracted from mouse tails showed that strain 76 had 18–42 (30.2+/−11.8), strain 79 had 31–45 (37.4+/−7.1), and strain 138 had 6–12 (8.0+/−4.0) copies of the *Flag-Dmp1α* transgene. Thus strains 76 and 79 were designated as high copy number *MDTG* strains, while strain 138 was low copy number strain.

**Figure 1:**
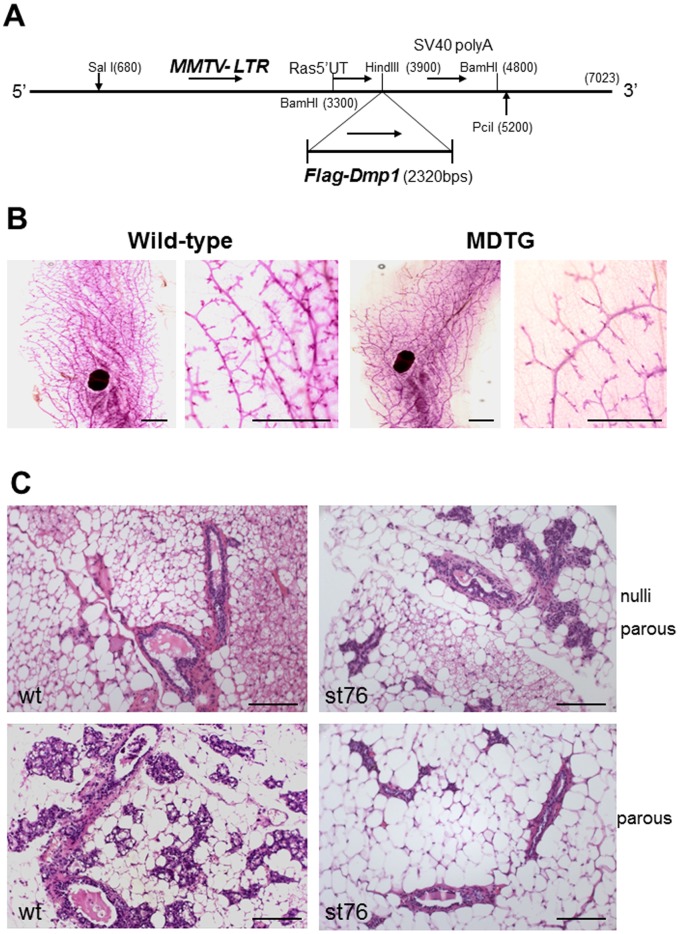
Creation of *MMTV-Flag-Dmp1α* mice. A. The *Flag-Dmp1* cDNA (*Bam*HI fragment) was recovered from the pFLEX1-Dmp1 vector and was cloned into the *Hind*III site of the transgenic vector with *MMTV* promoter (*MMTV-SV40-BSSK*). B. Whole mammary gland mounts from 12-week-old nulliparous *MDTG* (strain 79) and wild-type mice. Scale bars show 2 mm. C. Photomicrographs of nulliparous and multi-parous female mammary glands (MMG) from wild-type and strain 76 *MMTV-Flag-Dmp1* (*MDTG*) mice (12-week-old). Wild-type mammary glands were well-dilated with milk production, which became more prominent after getting pregnant. *MDTG* mammary glands, on the other hand remained small, unopened with little milk production. These mammary glands did not open well with very low milk production even after getting pregnant, making it difficult to feed their pups. The scale bars show 100 µm.

Compared to wild-type mice, the *MDTG* females showed significantly reduced (∼50% less) number of mammary glands and branching with poor alveolar sac development throughout the body, although the size of the germinal center remained the same (Fig. 1B). When stained with H&E, mammary glands (MMGs) from *MDTG* mice showed small and undilated tubules with little milk production in comparison to those of normal controls in null-parous mice (Fig. 1C, upper panels). The morphological difference of MMG between wild-type and *MDTG* mice was more prominent in parous females; they were well-branched and dilated with milk production in the former, but unopened or partially opened with little milk production in the latter (Fig. 1C, lower panels). Consistent with these findings, approximately 60% of *MDTG* parous females were unable to support their pups while wild-type FVB females were able, suggesting that MMG from *MDTG* females had defective function.

### Tissue-specific Expression of Flag-Dmp1 mRNA and Protein in Mammary Tissues

**Figure 2:**
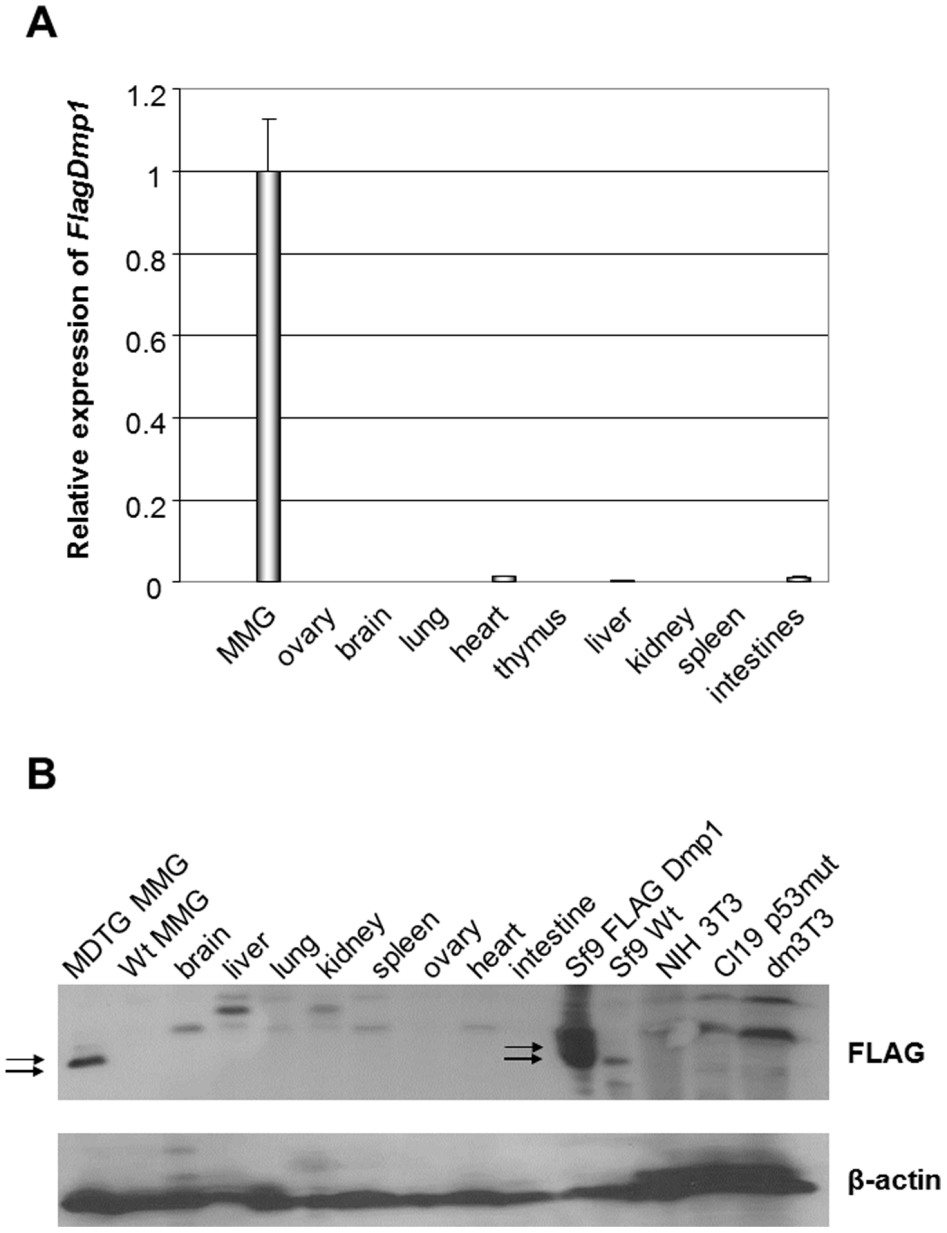
Expression of Flag-Dmp1 mRNA and proteins in tissues of *MDTG* mice. A. Expression of *Flag-Dmp1* mRNA in tissues from a mouse of *MDTG* (12-week-old, strain 76) as studied by real-time PCR. The level of *Flag-Dmp1* in lactating mammary glands was defined as 1.0. B. Expression of the Flag-Dmp1 protein in tissues from a *MDTG* mouse (12-week-old, strain 76). Proteins were extracted in ice-cold EBC buffer (19) and 100 µg of proteins were analyzed by Western blotting with the antibody to the FLAG tag.

Gene expression studies as demonstrated by real-time PCR showed tissue-specific expression of transgenic *Flag-Dmp1* mRNA in MMG from *MDTG* mouse strain 76 in comparison to other tissues (ovary, brain, lung, heart, thymus, liver, kidney, spleen, and intestines) from this transgenic mouse (Fig. 2A). We also analyzed the expression of the Flag-Dmp1 protein in tissues from *MDTG* mouse strain 79 and found tissue-specific expression of the Flag-Dmp1 protein in MMG from this transgenic mouse strain, confirming the selectivity of the promoter (Fig. 2B).

### Expression of the Flag-Dmp1 Protein Decreased Cell Proliferation in MMG from *MDTG* Mice

**Figure 3:**
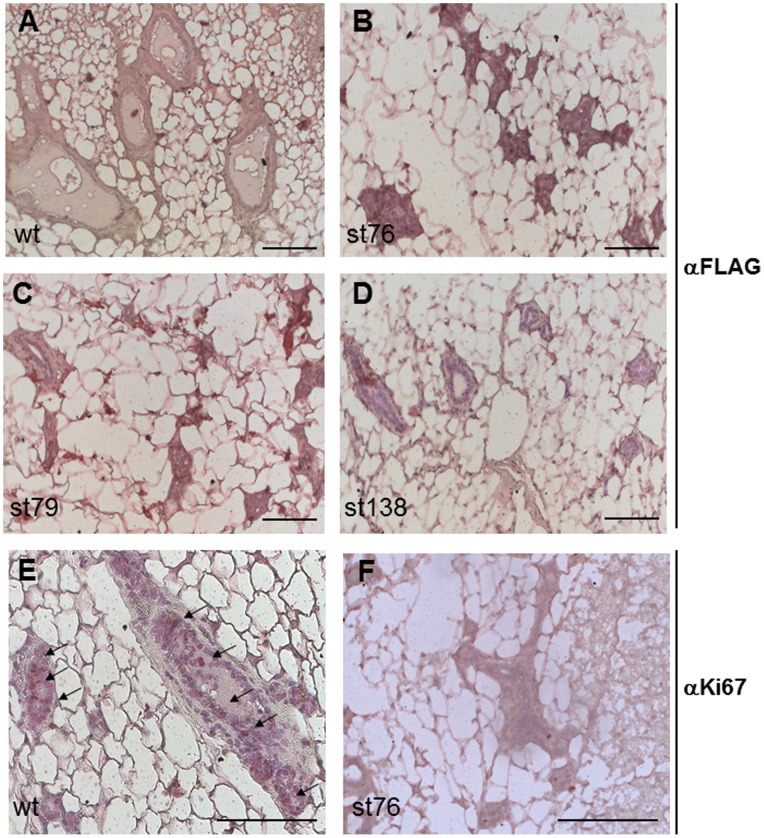
Immunohistochemical detection of Flag-Dmp1 and Ki67 in mammary glands of *MDTG* mice. A, B, C, and D. Immunohistochemical detection of the Flag-Dmp1 protein in nulliparous transgenic MMG. A) wild-type, B) *MDTG* strain 76, C) *MDTG* strain 79, and D) MDTG strain 138. E, F. Immunohistochemical detection of Ki67 in nulliparous MMG from wild-type (E) and *MDTG*, strain 76 (F) female. Note that Ki67 signals were barely detectable in MMG from a *MDTG* mouse.

In immunohistochemical (IHC) studies using an antibody to Flag, we detected significant Flag-Dmp1 signals in mammary glands from all three strains of *MDTG* mice (Fig. 3B–D), but only background signals were observed in wild-type MMG (Fig. 3A). The Flag-Dmp1 intensity in MMG from strains 76 and 79 mice was higher than that from strain 138, reflecting *Flag-Dmp1* transgene copy number differences among these strains. When blotting these tissues with an antibody for Ki67, a marker for cell proliferation, we detected 5–10 positive cells per MMG from wild-type nulliparous females but barely detected any in *MDTG* mice (Fig. 3E, F). The same patterns were obtained in females regardless of their age. These data suggest that MMG of *MDTG* remained unopened throughout their life due to strikingly decreased cell proliferation.

#### Induction of Ink4a/Arf proteins in MMG from *MDTG* mice

**Figure 4:**
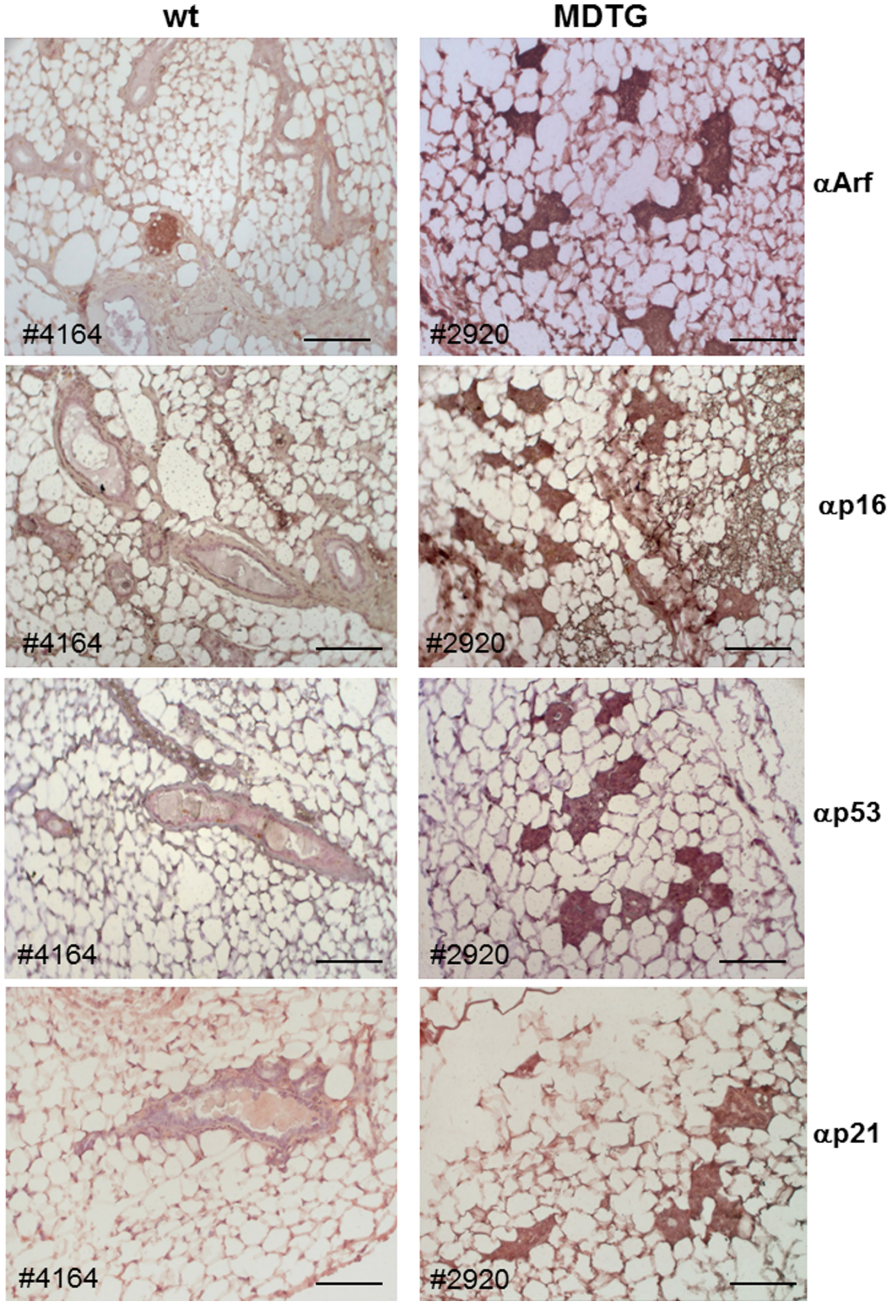
Immunohistochemical detection of p19^Arf^, p16^Ink4a^, p53, and p21^Cip1^ in mammary glands of *MDTG* mice. All of these proteins were induced in MMG from *MDTG* females suggesting the activation of the p53 and Rb pathways by Dmp1 *in vivo*. Scale bars show 100 µm.

Our recent studies indicate that both p19^Arf^ and p16^Ink4a^ are direct transcriptional targets of Dmp1α [39]. To determine the mechanism of low Ki67 expression, we stained MMG from *MDTG* mice with antibodies to Arf, Ink4a, p53, and p21^Cip1^ (Fig. 4). The induction of Arf, Ink4a, and p21^Cip1^ mRNAs in the MMG from *MDTG* mice were confirmed by real-time PCR (230–1,025 folds for *Arf*; 2,500–28,400 folds for *Ink4a*, and 57–115 folds for *p21*). These data indicate that both p53 and Rb pathways were strongly activated *in vivo* when Dmp1 is highly expressed in MMG (Fig. 4). There was no difference in estrogen receptor α (ERα) and progesterone receptor (PR) expression between WT and *MDTG* mice (Fig. S2). We also found that the EGF ligand amphiregulin (Areg) [40] level was not significantly affected in MMG from *MDTG* mice while both EGF and TGFα were upregulated (Fig. S3), indicating that the regulation of Areg by Dmp1 [41] is tissue-dependent. The data indicate that high expression of Ink4a and Arf proteins, not lack of hormonal receptors or EGF ligands, contributed to the poor MMG development in *MDTG* females (Fig. 3).

### HER2/Neu-driven Mammary Carcinogenesis is Significantly Delayed in *MDTG;neu* Double Transgenic Mice

**Figure 5:**
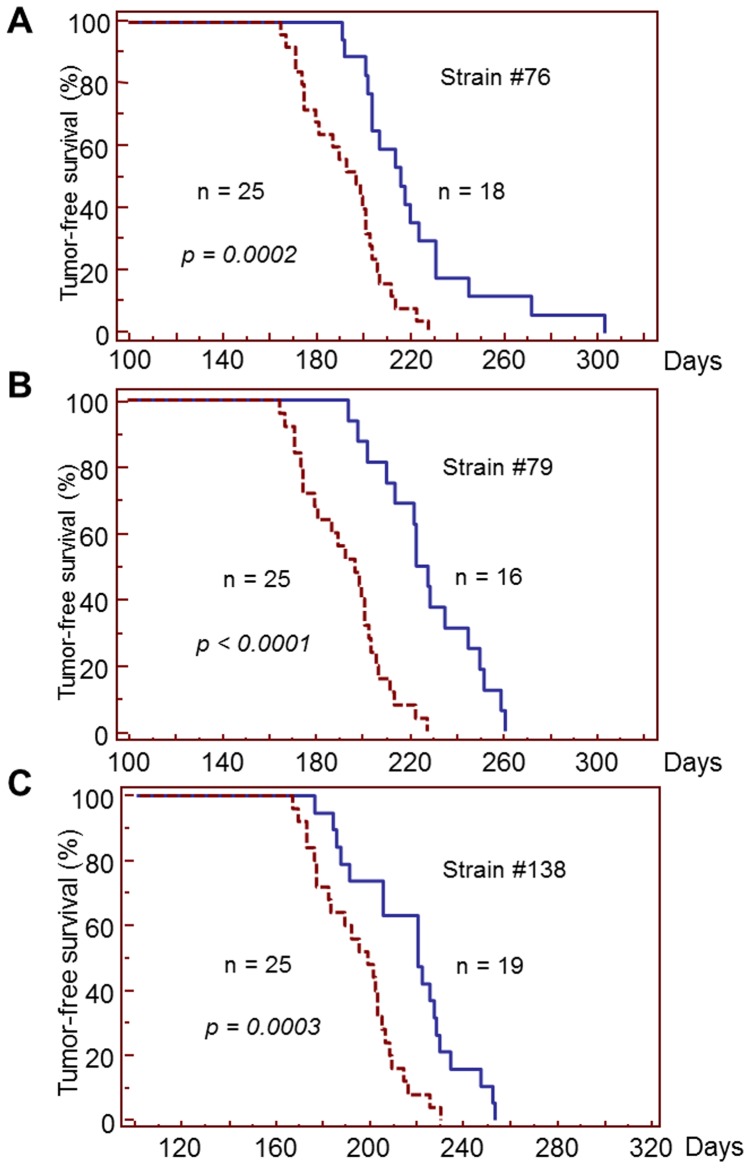
Disease-free survival (DFS) curves of *MDTG;neu* mice in each strain. DFS curves for each strain of *MDTG;neu* females are shown. The *p* values were calculated in comparison to the survival of wild-type *MMTV-neu* mice (n = 25; medium survival, 197 days).

**Figure 6:**
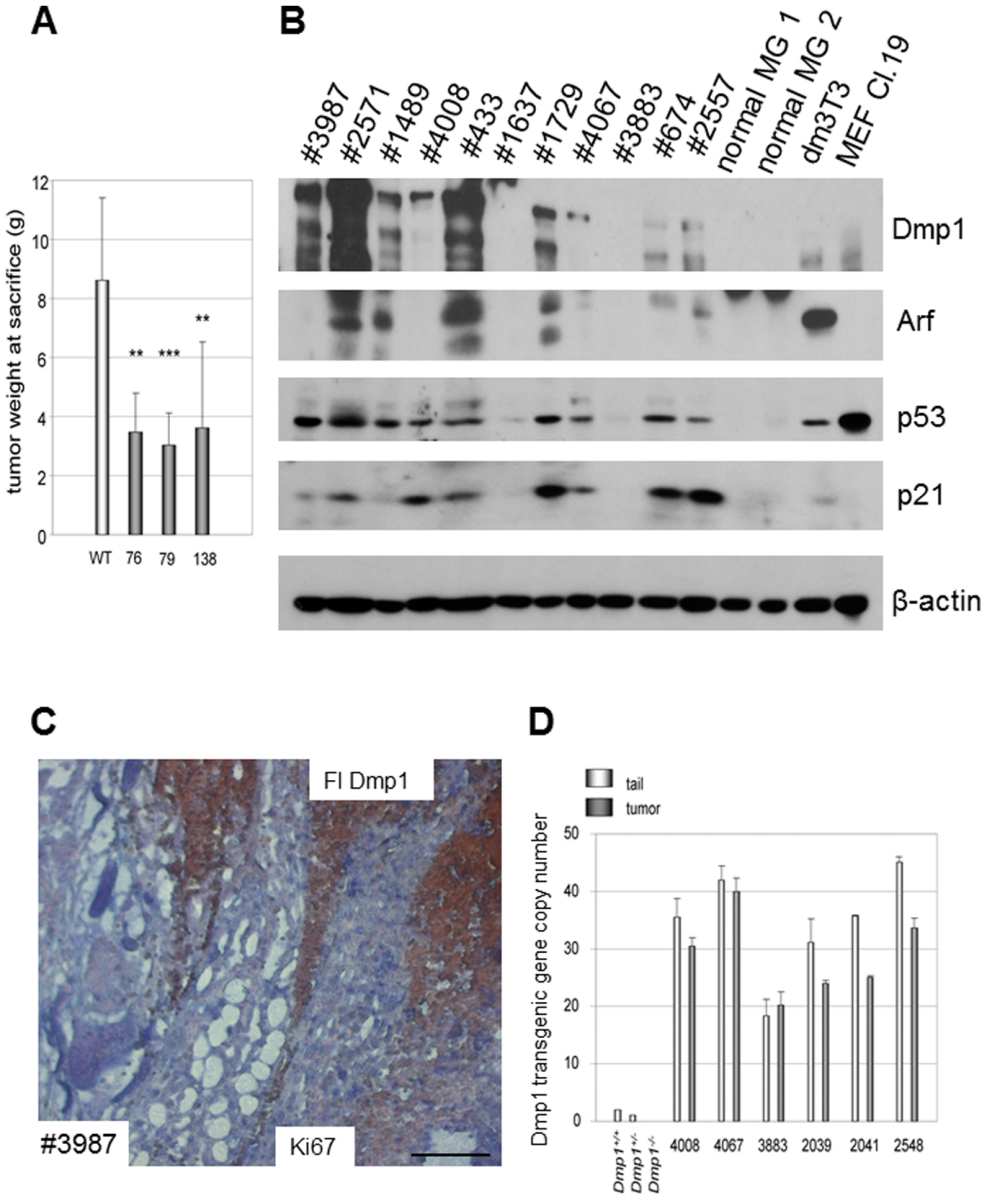
Mutually exclusive expression of Flag-Dmp1 and Ki67 in *MDTG;neu* tumors. A. Total tumor weight harvested at sacrifice. The amount of tumors recovered at sacrifice (4 weeks after the first tumor was found) was significantly decreased in all three transgenic strains. B. Western blotting analyses of proteins expressed in *MDTG;neu* tumors. C. Mutually exclusive expression of Flag-Dmp1 and Ki67 in a *MDTG;neu* tumor (strain 76, #3987). Flag-Dmp1 was stained brown; Ki67 was stained blue. Scale bars show 100 µm. D. Gene copy number assay for *Flag-Dmp1* in paired tails and *MDTG;neu* tumors showing partial deletion of the transgene in half of the cases.

Our previous study showed that *MMTV-neu*-induced mammary tumorigenesis was significantly accelerated in *Dmp1*-deficient mice, indicating a critical role of Dmp1 as a mediator of aberrant HER2 signaling and the Arf-p53 pathway [11]. To determine the effect of high Dmp1α expression in mammary carcinogenesis by HER2/neu, we crossed *MMTV-neu* mice with *MDTG* mice. The generated 16–19 *MDTG;neu* bitransgenic female mice still developed mammary tumors; however, compared to *MMTV-neu* mice, *MDTG;neu* bitransgenic mice from all three strains exhibited significantly delayed tumor formation (Fig. 5). The median tumor/disease-free survival (DFS) for the *MMTV-neu* mice was 197 days (n = 25), but it was significantly extended in *MDTG;neu* mice to 216 days (from strain 76; n = 18, *p* = 0.0002), 223 days (from strain 79; n = 16, *p*<0.0001), and 211 days (from strain 138, n = 19; *p = *0.0003). The average amount of tumors harvested at 4-week time point after the first tumor appearance was significantly decreased in bitransgenic mice from all three strains (*MMTV-neu*, 8.61+/−2.8 g (n = 10) vs. 3.48+/−1.32 g (n = 6, *p = *0.0010) for strain 76; 3.08+/−1.08 g (n = 11, *p*<0.0001) for strain 79; 3.63+/−2.89 g, (n = 9, *p* = 0.0014) for strain 138) (Fig. 6A). Thus, not only was DFS extended, but also the absolute amount of tumors present when they died were decreased in *MDTG;neu* bitransgenic mice in comparison to *non-MDTG*;*neu* mice. These data suggest that transgenic expression of Dmp1α has significant effects on preventing HER2/neu-driven mammary carcinogenesis.

### Analysis of the p53 and Rb Pathways in Dmp1-resistant Tumors from *MDTG; neu* Mice

Although Dmp1 showed a significant role in inhibiting *MMTV-neu*-induced mammary carcinogenesis, all *MDTG;neu* mice died of tumors 1–2 months later than *non*-*MDTG*;*neu* transgenic mice. Thus, we investigated whether there was any molecular events that occurred in *MDTG;neu* mice during the course of tumor development. Western blot analyses for the MMG of *MDTG;neu* animals demonstrated that 2 of 11 tumors had high Flag-Dmp1 expression (#2571, #433), three had medium Flag-Dmp1 expression (#3987, #1489, #1729), and four had low expression, while Flag-Dmp1 was barely detectable in two tumors (#1637, #3883) (Fig. 6B, top). The Arf and p53 proteins well correlated with Flag-Dmp1 expression, suggesting that the Dmp1-Arf-p53 signaling was still functional in *MDTG;neu* tumor cells. The p21^Cip1^ levels correlated with Flag-Dmp1 levels except for the two tumors (#674, #2557), suggesting some other protein(s) induced p21^Cip1^ in these cases.

**Figure 7:**
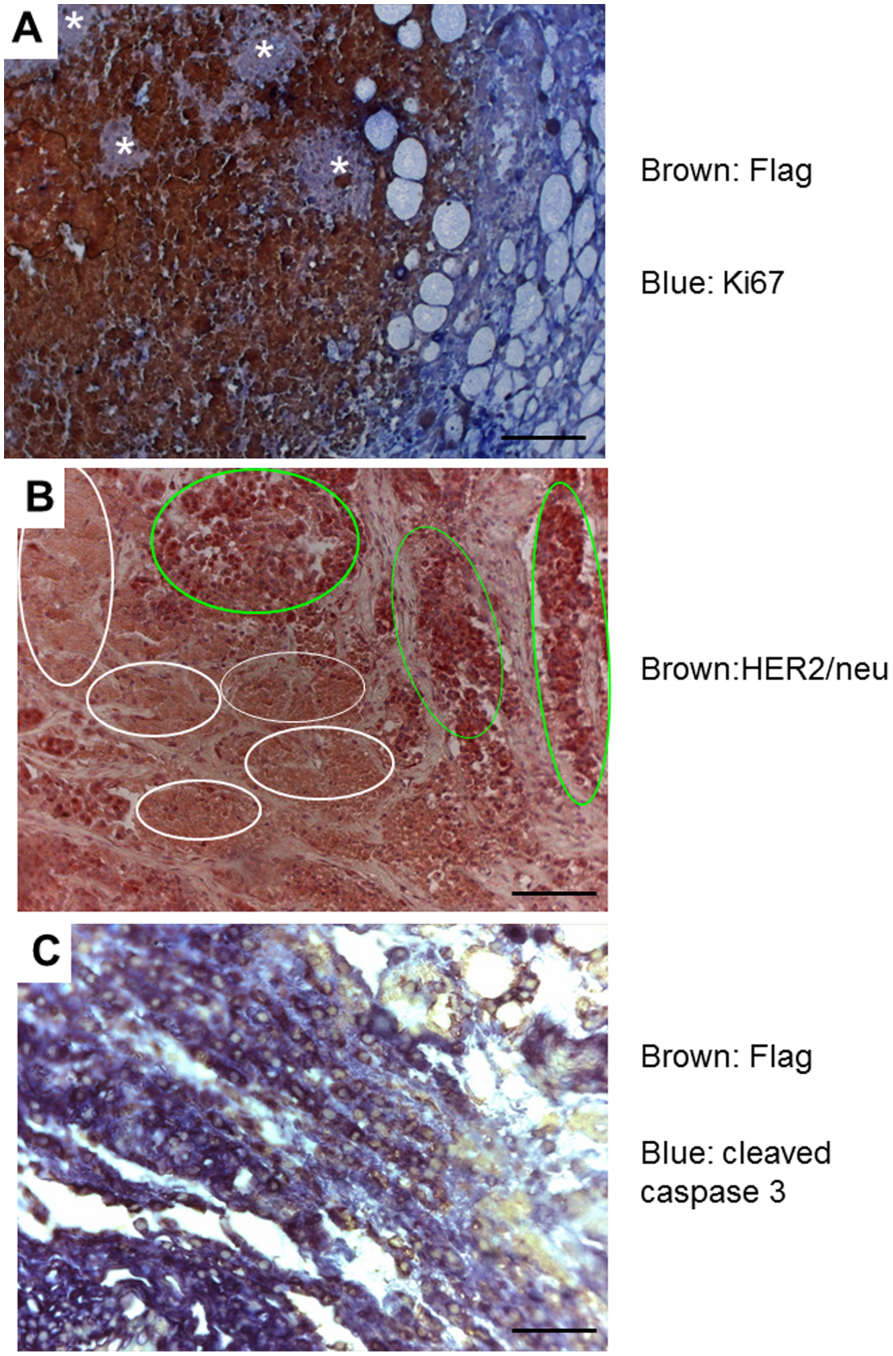
Immunohistochemical studies of *MDTG;neu* tumors. A. A *MDTG;neu* tumor (#3883) that shows areas of Flag-Dmp1 (brown) and islands of Dmp1-resistant tumor growth (white arrows). The Ki67-positive area is stained blue. B. Immunohistochemical staining of *MDTG;neu* tumor (#3987) with HER2 antibody. All of the tumor cells express the HER2/neu protein. Light green circles show areas of tumor growth while white circles show areas of tumor cell death. C. Double immunohistochemical staining of a *MDTG;neu* tumor (#3883) that shows significant overlap of Flag-Dmp1-positive cells (stained light brown in nuclei) and cleaved-caspase 3-positive cells (blue in the cytoplasm). A, B, C. Scale bars show 100 µm.

We then studied the cell proliferative status of *MDTG;neu* tumors. Double staining of *MDTG;neu* mammary tumors with Flag (brown) and Ki67 (blue) antibodies showed mutually exclusive pattern of their expression (Fig. 6C). This suggests that either cells expressing Flag-Dmp1 stopped proliferating or only tumor cells that succeeded in downregulating Flag-Dmp1 protein were growing. Consistently, we observed numerous areas of Ki67(+) Flag-Dmp1(−) cells (shown in stars, Fig. 7A), supporting our hypothesis that inactivation of the *Flag-Dmp1* transgene is necessary for tumor cell proliferation. To further investigate the mechanisms of decreased Flag-Dmp1 expression in *MDTG;neu* tumors, we extracted genomic DNA from matched tail and tumor samples and calculated *Flag-Dmp1* gene copy numbers by real-time PCR (Fig. 6D). The results showed that the transgenic *Flag-Dmp1* was retained only in half of the *MDTG;neu* tumors (#4008, #4067, and #3883) and there was partial (20–25%) loss of the transgene in the other half of tumors. This is possibly because some HER2/neu-expressing cells that stochastically deleted the *FlagDmp1* gene began to proliferate *in vivo* and emerged as tumors since the protein was interfering with neoplastic cell proliferation. We also observed that the *Flag-Dmp1* mRNA was downregulated in some tumors in which the transgene was not deleted (data not shown). Thus, we conclude that both loss of the *Flag-Dmp1* gene and downregulation of Flag-Dmp1 mRNA or protein contributed to generation of highly proliferative lesions, allowing HER2/neu tumors to progress.

Finally, we stained tumors from *MDTG;neu* mice with an antibody to neu. We found that all tumors were positive for neu (Fig. 7B) with some areas showing stronger signals (light green circles) than the others (white circles), suggesting that significant areas of *MDTG;neu* tumor was in the process of cell death. To confirm this prediction, we double stained *MDTG;neu* tumors with antibodies to the Flag (brown) and cleaved caspase 3 (blue) (Fig. 7C). The Flag and caspase 3 staining signals were overlapping, indicating that Flag-Dmp1-positive area was undergoing apoptosis. Thus, high expression of Flag-Dmp1 induces the cell death of early stage *neu* tumors.

## Discussion

In this study, we created transgenic mice that have tissue-specific expression of the *Dmp1α* gene in mammary glands, driven by the *MMTV* promoter. None of the transgenic females, neither nulliparous nor mono−/multi-parous, exhibited any malignant transformation within two years of observation period. The same observation was made also in males. The mammary glands in the females remained small and unopened with markedly reduced milk production in all cases, and expressed senescence markers such as Arf, p16, p21, and p53. Our data indicate that Dmp1 inhibits HER2/neu-mediated carcinogenesis *in vivo*. Since *Arf*, p*16*, and *p53* themselves are tumor suppressor genes, they must have contributed to the significantly delayed mammary carcinogenesis in the *MDTG;neu* bitransgenic mice.

Previous studies suggest that the Arf gene is mostly activated by oncogenes, such as mutant Ras, HER2, E1A, v-abl, Myc, and E2F-1 [22], [42]. Thus, Arf is a major tumor suppressive molecule that diverts hyperproliferative signals to p53-mediated cell cycle arrest and/or apoptosis. As an activator of Arf, Dmp1 is unique in its role of achieving cell cycle arrest/senescence without causing cellular transformation in either cultured cells or *in vivo*. Our published studies have shown that the senescence response induced by mutant Ras or HER2 is mediated by Dmp1, while increased Myc or E2F-1 expression downregulates Dmp1 [27]. This suggests that Dmp1 is a mediator to convey oncogenic signals to the p53 pathway if they induce cell cycle arrest/senescence, but does not mediate the hyperproliferation signals for apoptosis, such as from Myc, E2F-1. We previously demonstrated that the *Dmp1* promoter can be activated by TNFα, an endogenous pyrogen that induces fever, apoptotic cell death, and cachexia, and inhibits tumorigenesis [29]. Our recent study showed that the Dmp1 protein was induced *in vivo* in response to doxorubicin [24]; thus Dmp1 likely receives signals from sources other than oncogenes to activate both p53 and Rb pathways via Arf [21] and p16^Ink4a^ [39]. It is currently unclear whether Dmp1 is involved in hypoxic response, osmotic pressure, malnutrition signaling, or hormonal signaling. The presence of 16 potential phosphorylation sites on Dmp1 raises the possibility of Dmp1 regulation via phosphorylation. Thus, further studies will be needed to elucidate the signaling pathways that lead to increased Dmp1 expression and activity.

We observed significant increase of DFS of *MDTG;neu* mice compared to *MMTV-neu* mice. This happened when *MMTV-neu* mice were crossed with all three *MDTG* transgenic founder lines. Interestingly the *p* values of the DFS were the smallest in strain 79 that had highest *Flag-Dmp1α* gene copy numbers: followed by strain 76 (high copy), and strain 138 (low copy), suggesting that the levels of Flag-Dmp1α transgene/protein expression matters for Dmp1 to show its biological effects. Dmp1 not only delayed HER2/neu-driven mammary carcinogenesis, but also decreased tumor volumes when the animals were sacrificed (Fig. 6A). Again the tumor-reducing effect was highest in strain 79, lowest in strain 138, suggesting that the effect of Dmp1 is dose-dependent. However it should be noted that mammary tumors arose later, regardless of *Flag-Dmp1α* copy numbers and the differences of transgene integration sites dependent on the strain. In our IHC studies, we observed that the Flag-Dmp1α and Ki67 protein expression were mutually exclusive, suggesting that Flag-Dmp1-positive areas were not proliferating and thereby preventing tumor progression *in vivo*. Moreover, we saw significant overlap between Flag-Dmp1-positive areas and cleaved caspase 3(+) areas in *MDTG;neu* tumors (Fig. 7C), indicating that Flag-Dmp1α induces apoptotic cell death *in vivo*. When we studied how the *MDTG;neu* animals eventually developed tumors, we observed reduced Flag-Dmp1 protein expression in *MDTG;neu* tumors in nearly 80% of cases. Partial loss of the *Flag*-*Dmp1* transgene in tumors or downregulation of the *Flag-Dmp1* mRNAs or proteins could contribute to this observation. The lack of correlation between the mRNA and protein levels of Flag-Dmp1 (data not shown) indicates that Flag-Dmp1 downregulation occurred at either transcriptional or protein level in the tumors of *MDTG;neu* mice. Indeed, we saw focal proliferation of HER2/neu tumors in islands lacking Flag-Dmp1 expression (Fig. 7A). Whether Dmp1 protein undergoes ubiquitin-mediated degradation remains to be determined.

In a related study, Yang *et al*. [43] created *MMTV-p16^INK4a^* transgenic mice to study the effects of p16^INK4a^ on ErbB2-induced mammary tumorigenesis using *MMTV-ErbB2* (wild-type; WT) rather than *MMTV-neu* (mutant) used in this study. The *p16^INK4a^* transgene dramatically delayed mammary tumorigenesis by ErbB2, indicating that ErbB2-mediated deregulation of cyclin D1/Cdk4/6 is a crucial step of tumor formation. Although p16^INK4a^ might have a more prominent role than Flag-Dmp1 in preventing mammary carcinogenesis based on our results and the theirs, the difference can be simply attributed to the fact that mild wild-type ErbB2 mouse model [17], [43] was used by this group while ErbB2 mutant model [16] was used by us.

Our study shows highly expressed Dmp1 induces Ink4a/Arf, p53, and p21 *in vivo*. It has been reported that both *Ink4a* and *Arf* levels markedly increase in almost all rodent tissues with advanced age, while there is little change of other Cdk inhibitors such as p15, p18, and p19 [44]. This increase occurred in both epithelial and stromal cells of different lineages. This aging-related induction of *Ink4a/Arf* was attenuated in organs by caloric restriction with diminished expression of senescence markers, indicating that *Ink4a/Arf* expression themselves are biomarkers of aging [44]. Since Dmp1 does not have any oncogenic activity, we hypothesize that Dmp1 is also a biomarker of aging that transmits relevant signals to its downstream *Ink4a/Arf genes.* Whether Dmp1 is a *bona fide* regulator of aging needs to be addressed in the future by checking the expression of *Ink4a/Arf* and other Cdk inhibitors in tissues from *Dmp1* wild-type and deficient mice.

Garcia-Cao *et al*. [45] established and characterized mice carrying supernumerary copies of the wild-type *p53* gene as a large genomic transgene. These “super *p53*” mice, which carried *p53*-transgene alleles in addition to the two endogenous *p53* alleles, exhibited an enhanced response to DNA damage, and importantly, were protected from cancer when compared to normal mice. It should be noted that constitutive activation of p53, such as chronic exposure to stress, could result in accelerated aging as demonstrated by Tyner *et al* [46]. In contrast, “super *p53*” mice showed a normal aging process despite having clearly increased p53 activity. A critical feature of the “super p53” mice is that the basal levels of p53 activity are not affected. This is important because further increase in gene dosage of p53 might eventually reach a threshold at which deleterious effects would be noticeable, such as defective tissue regeneration, growth atrophies, and premature aging. This suggests that increases in normally regulated p53, as in the “*super p53*” mice, could confer cancer protection without affecting aging, while constitutively high levels of active p53 or expression of a truncated carboxyl-terminal p53 promotes aging [44], [45]. Thus, cancer resistance could be enhanced by a simple genetic modification of p53 in the absence of undesirable effects. Likewise, Mathew *et al*. [47] generated a “super *Ink4a/Arf*” mouse strain carrying a transgenic copy of the entire *Ink4a/Arf* locus and demonstrated that modest increase in the activity of the *Ink4a/Arf* tumor suppressors also resulted in a beneficial cancer-resistant phenotype without affecting normal viability or aging. Likewise, it will be possible to prevent cancers by stimulating the Dmp1 activity or increasing its protein levels/activity without causing toxic effects of accelerated aging.

Four distinct stages of the breast epithelial cells are defined on the basis of the estrogen receptor profile. These are: (i) prepuberty, when both ERα and ERβ are present; (ii) pregnancy, when ERβ is present in the majority of epithelial cells, ERα-expression is low, and few cells express both receptors; (iii) lactation, when ERα and ERβ are both expressed in the majority of epithelial cells; and (iv) post lactation, when ERα is extremely low and there is little co-localization of the ERα/β receptors [48]. In mammary glands of the *MDTG* mice, ER or PR level did not decrease in comparison to that in wild-type. Collaborative roles of EGF receptor (EGFR) ligands in mammopoiesis and lactogenesis have been reported [40]. It was shown that triple knockout mice for the three EGFR ligands for EGF, Areg, and TGFα showed neonatal lethality due to the absence of milk production of the mother [40]. In the MMG of *MDTG* females, Areg expression did not significantly change while both EGF and TGFα were upregulated. We therefore conclude that markedly reduced proliferation of mammary epithelial cells in *MDTG* females were due to the Dmp1-mediated Ink4a/Arf overexpression rather than downregulation of hormonal receptors or EGF ligands.

In conclusion, we have established transgenic mice that specifically express the *Dmp1α* gene in the mammary glands. These animals show tumor suppressive effects on HER2/neu-driven tumorigenesis by inducing cell cycle arrest and/or apoptosis. These transgenic mice will be useful to predict tumor-suppressive effects of Dmp1 in other transgenic mice that are prone to mammary tumor development, and identify novel Dmp1 target genes/proteins *in vivo*. Molecules that specifically activate the *Dmp1* promoter or the Dmp1α protein will be effective novel chemotherapeutic agents to induce regression of tumor growth *in vivo*.

## Supporting Information

Figure S1 **PCR-mediated amplification of the cloned cDNA using transgenic mouse tails.** The results from strains 76 and 79 are shown.
(TIF)

Figure S2 **Immunohistochemical staining of a wild-type (#4164) and MDTG (#2920, strain 79) MMG for hormone receptors.** ER: estrogen receptor; PR: progesterone receptor.
(TIF)

Figure S3 **Immunohistochemical staining of a wild-type (#4164) and MDTG (#2920, strain 79) MMG for EGFR ligands.** Areg: Amphiregulin; EGF: Epidermal Growth Factor; TGFβ: Transforming Growth Factor beta.
(TIF)
