# Supplementary figures and images for: Dmp1α Inhibits HER2/neu-Induced Mammary Tumorigenesis
Source: PLoS One. 2013 Oct 29;8(10):e77870. doi: 10.1371/journal.pone.0077870 (PMC3812138; doi:10.1371/journal.pone.0077870)

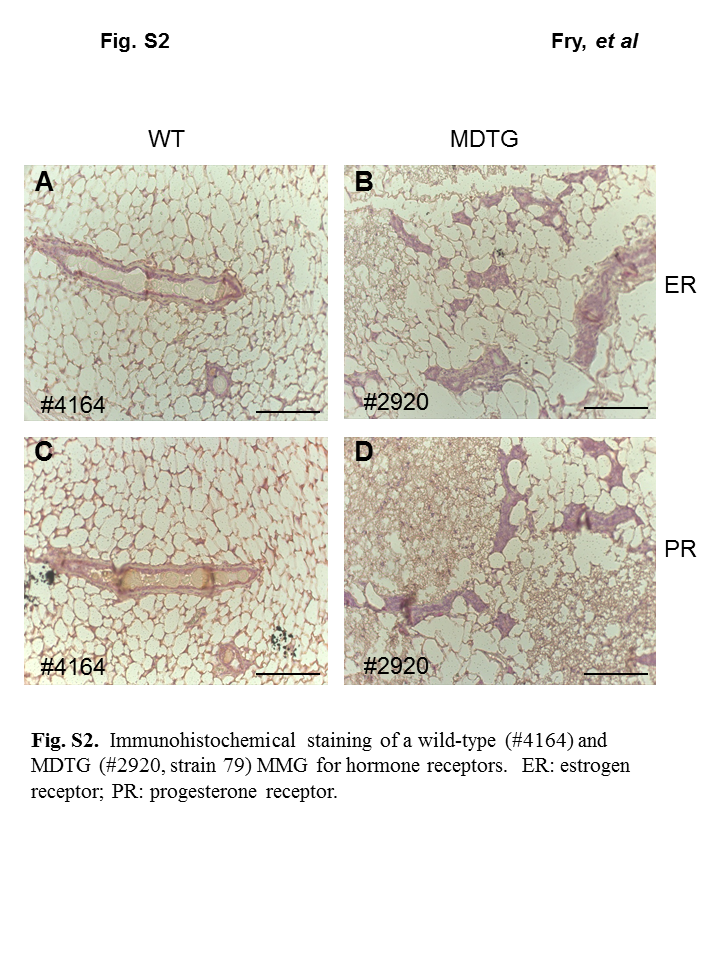

Supplement: Figure S2 — Immunohistochemical staining of a wild-type (#4164) and MDTG (#2920, strain 79) MMG for hormone receptors. ER: estrogen receptor; PR: progesterone receptor. (TIF) [file pone.0077870.s002.tif]

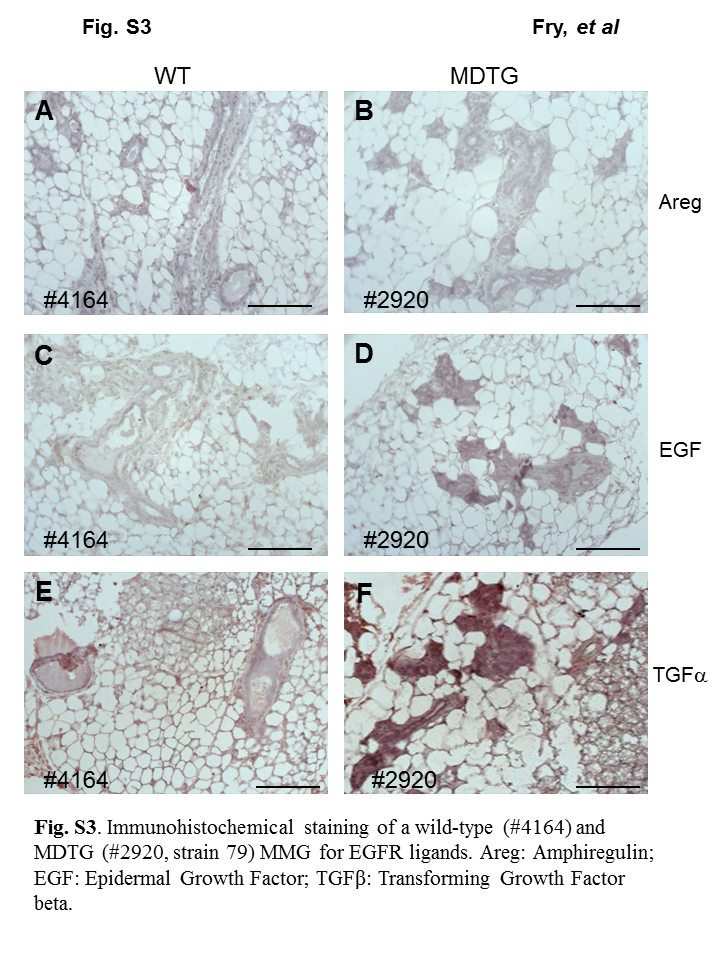

Supplement: Figure S3 — Immunohistochemical staining of a wild-type (#4164) and MDTG (#2920, strain 79) MMG for EGFR ligands. Areg: Amphiregulin; EGF: Epidermal Growth Factor; TGFβ: Transforming Growth Factor beta. (TIF) [file pone.0077870.s003.tif]
